# Sexually dimorphic metabolic effects of a high fat diet on knee osteoarthritis in mice

**DOI:** 10.1186/s13293-024-00680-6

**Published:** 2024-12-05

**Authors:** Timothy M. Griffin, Erika Barboza Prado Lopes, Dominic Cortassa, Albert Batushansky, Matlock A. Jeffries, Dawid Makosa, Anita Jopkiewicz, Padmaja Mehta-D’souza, Ravi K. Komaravolu, Michael T. Kinter

**Affiliations:** 1 Aging and Metabolism Research Program, Oklahoma Medical Research Foundation, Oklahoma City, OK 73104 USA; 2 Veterans Affairs Medical Center, Oklahoma City, OK 73104 USA; 3 University of Oklahoma Health Sciences Center, Oklahoma City, OK 73104 USA; 4 Arthritis and Clinical Immunology Research Program, Oklahoma Medical Research Foundation, Oklahoma City, OK 73104 USA; 5 Present Address: Labcorp Drug Development, Indianapolis, IN USA; 6 Present Address: VA Oklahoma City Health Care, Oklahoma City, OK USA; 7 Present Address: Ilse Katz Institute for Nanoscale Science and Technology, Ben-Gurion University of the Negev, Be’er Sheva, 84105 Israel; 8 Present Address: University of Western Australia, Perth, Western Australia, Australia; 9 Present Address: Panier Group, Max Planck Institute for Biology of Ageing, Joseph-Stelzmann-Strasse 9B, 50931 Cologne, Germany; 10 Present Address: Immunology Center of Georgia, Augusta University, Augusta, GA 30912 USA

**Keywords:** Sex differences, Osteoarthritis, Obesity, Metabolic syndrome, High fat diet, Gut microbiome, Inflammation, Infra-patellar fat pad, Cartilage

## Abstract

**Background:**

Women have a higher risk of developing osteoarthritis (OA) than men, including with obesity. To better understand this disparity, we investigated sex differences in metabolic and inflammatory factors associated with OA using a diet-induced mouse model of obesity. We hypothesized that 20 weeks of high-fat diet (HFD) would induce sexually dimorphic changes in both systemic and local risk factors of knee OA.

**Methods:**

Male and female C57BL/6J mice were fed Chow or HFD from 6 to 26 weeks of age (*n* = 12 per diet and sex). We performed broad metabolic phenotyping, 16 S gut microbiome analysis, targeted gene expression analysis of synovium-infrapatellar fat tissue, targeted gene expression and proteomic analysis of articular cartilage, chondrocyte metabolic profiling, and OA histopathology. Two-way ANOVA statistics were utilized to determine the contribution of sex and diet and their interaction on outcomes.

**Results:**

Mice fed HFD weighed 1.76-fold (*p* < 0.0001) and 1.60-fold (*p* < 0.0001) more than male and female Chow cohorts, respectively, with both sexes reaching similar body fat levels (male: 43.9 ± 2.2%; female: 44.1 ± 3.8%). HFD caused greater cartilage pathology (*p* < 0.024) and synovial hyperplasia (*p* < 0.038) versus Chow in both sexes. Cartilage pathology was greater in male versus female mice (*p* = 0.048), and only male mice developed osteophytes with HFD (*p* = 0.044). Both sexes exhibited metabolic inflexibility on HFD, but only male mice developed glucose intolerance (*p* < 0.0001), fatty liver (*p* < 0.0001), and elevated serum amylase (*p* < 0.0001) with HFD versus Chow. HFD treatment caused sex-dependent differences in gut microbiota beta diversity (*p* = 0.01) and alteration in specific microbiome clades, such as a HFD-dependent reduction in abundance of *Bifidobacterium* only in male mice. In knee synovium and infrapatellar fat tissue, HFD upregulated the expression of pro-inflammatory and pro-fibrotic genes predominantly in female mice. In cartilage, lipid metabolism proteins were more abundant with HFD in male mice, whereas proteins involved in glycolysis/gluconeogenesis and biosynthesis of amino acids were greater in cartilage of female mice. Sex-dependent metabolic differences were observed in cartilage from young, healthy mice prior to pubertal maturation, but not in primary juvenile chondrocytes studied in vitro.

**Conclusions:**

HFD induced numerous sex differences in metabolic and inflammatory outcomes, especially in joint tissues, suggesting that sex-specific cellular processes are involved during development of early-stage OA with obesity.

**Supplementary Information:**

The online version contains supplementary material available at 10.1186/s13293-024-00680-6.

## Highlights

High-fat diet caused osteoarthritis pathology in male and female mice, including cartilage degradation and synovial hyperplasia, with greater overall pathology and osteophyte development in male mice.

High-fat diet upregulated the expression of pro-inflammatory and pro-fibrotic genes in synovium and infrapatellar fat pad tissue, predominantly in female mice.

High-fat diet upregulated lipid metabolism proteins in cartilage of male mice, whereas proteins involved in glycolysis/gluconeogenesis and biosynthesis of amino acids were more abundant in cartilage of female mice regardless of diet.

These findings suggest that sex-specific cellular processes play a role in the development of early-stage osteoarthritis associated with obesity.

**Supplementary Information:**

The online version contains supplementary material available at 10.1186/s13293-024-00680-6.

## Plain language summary

Osteoarthritis is a painful and physically disabling synovial joint disease involving progressive degradation of articular cartilage, abnormal bone growth below and adjacent to the cartilage, and variable degrees of inflammation and fibrosis in joint connective tissues. Obesity increases the risk of osteoarthritis due to altered joint loading, increased inflammation, and associated metabolic disorders. Women are more likely to develop osteoarthritis than men, including in the context of obesity. However, the sex-specific factors that increase the risk of osteoarthritis in women are not well understood. We used a well-established mouse model of high-fat diet-induced obesity to investigate sex differences in metabolic and inflammatory risk factors associated with knee osteoarthritis. High-fat diet feeding caused cartilage damage and synovial thickening in both male and female mice, while only male mice developing altered bone growth adjacent to cartilage. Although high-fat diet feeding increased body fat to the same level in both sexes, only male mice developed insulin resistance and fatty livers. Conversely, in knee joint connective tissue, high-fat diet feeding increased the expression of genes that cause inflammation and fibrosis predominately in female mice. Additional sex differences were observed in the cartilage, with high fat diet increasing proteins involved with fat metabolism in male mice, whereas proteins important for carbohydrate and protein metabolism were more abundant in female mice. The numerous metabolic and inflammatory differences observed between male and female mice suggest that sex-specific cellular processes are involved in the development of early-stage osteoarthritis associated with obesity.

## Background

Although it is well established that women have a higher risk of developing osteoarthritis (OA) compared to men [1, 2], the cause of this disparity is not well understood due to the involvement of numerous social and biologic factors [3]. This topic was recently addressed in two comprehensive reviews, which advocated for more research on sex and gender differences in OA risk factors, disease etiology, symptoms, and treatment efficacy [4, 5]. In this regard, two recent population-based studies and a systematic review of sex differences in OA risk factors provided an intriguing new perspective on one of the strongest OA risk factors—obesity. The authors reported that the effect of obesity on the incidence of radiographic knee OA and total knee replacement was greater in women versus men [5–7]. Many obesity-associated factors contribute to the development of OA, including altered biomechanics, metabolic inflammation, and gut dysbiosis. As we recently reviewed, clinical studies suggest a sexually dimorphic role for adipose tissue and the adipokine leptin contributing to OA in women versus diabetes contributing to OA in men [8]. To better understand how obesity-associated metabolic diseases influence OA in a sex-dependent manner, the goal of our study was to identify sexually dimorphic effects of high-fat diet-induced obesity on systemic and local risk factors of knee OA in mice.

Pre-clinical studies with rodents have been instrumental in establishing a basic understanding of sexually dimorphic mechanisms of metabolic homeostasis and inflammation [9], and high fat diet (HFD) treatment in rodent models is widely used to study the pathophysiology of obesity and OA [10]. Unfortunately, many studies, including those from our lab [11–16], only utilized a single sex or did not explicitly seek to identify sex differences associated with OA [17]. Thus, despite the high clinical burden of obesity and OA, especially in women, there is limited insight into sex-dependent mechanisms linking obesity and knee OA in preclinical animal models. Here, we utilized one of the most widely studied rodent models of diet-induced obesity and OA: C57BL/6J mice fed a defined, 60% kcal fat diet. Based on our previous studies, we conducted high-fat feeding from 6 to 26 weeks of age to induce early-stage knee OA [11]. We performed broad systemic metabolic phenotyping, 16 S gut microbiome analysis, targeted gene expression analysis of synovium-infrapatellar fat tissue, targeted gene expression and proteomic analysis of articular cartilage, chondrocyte metabolic profiling, and OA histopathology. We hypothesized that 20 weeks on a HFD would induce sexually dimorphic changes in both systemic and local risk factors of knee OA, which may serve as a framework for future investigations of sex differences in the pathophysiology of obesity-induced OA.

## Methods

### Animals, diet treatments, and phenotyping

**Table 1:**
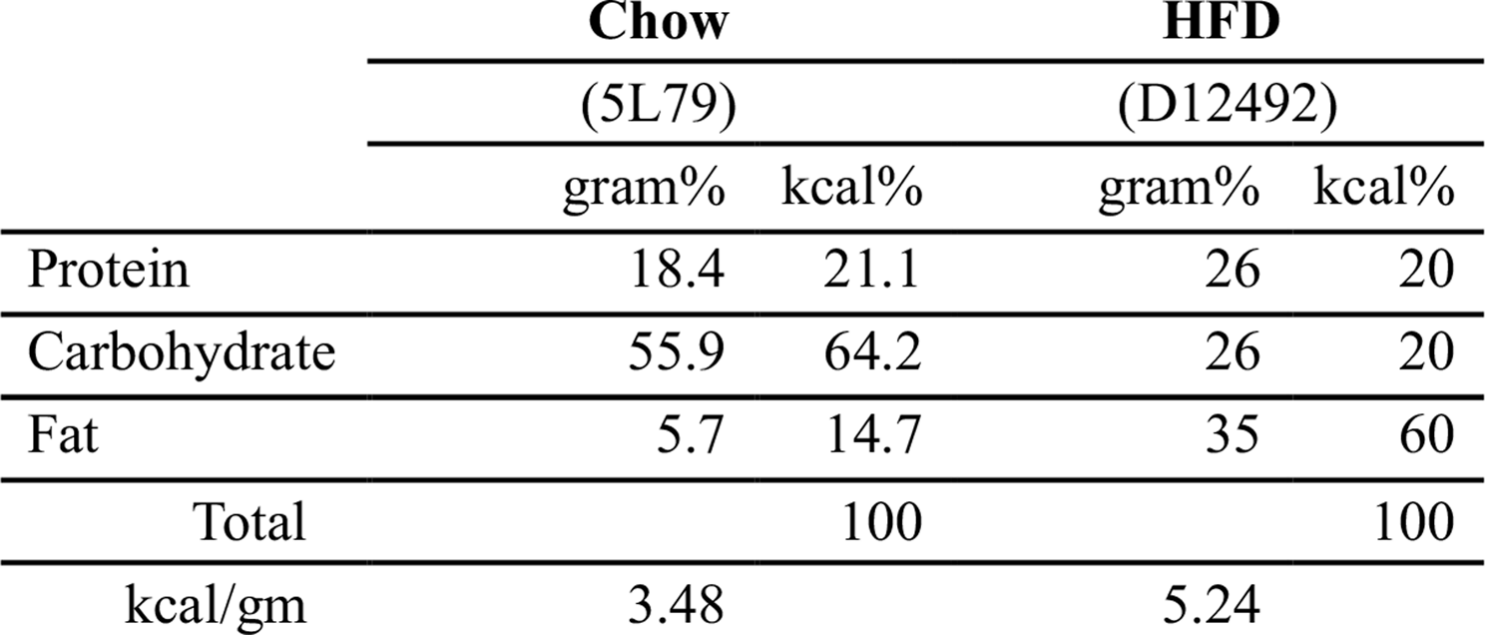
Macronutrient composition of diets

All experiments were conducted according to protocols approved by the AAALAC-accredited IACUC at the Oklahoma Medical Research Foundation (protocols 14–54 and 17–60). 4-week-old male and female C57BL/6J mice (*n* = 24 per sex) were purchased from The Jackson Laboratory (USA) in four cohorts of 12 animals over the course of six weeks. Mice were purchased as cohorts so that metabolic testing and euthanasia could be performed at consistent times of day to minimize potential circadian-related variation. Mice were group housed in a specific-pathogen free facility under a controlled environment (22 ± 3 °C on 14 h:10 h light/dark cycles, lights on at 06:00) in ventilated cages (3–4 animals/cage) with *ad libitum* access to chow (LabDiet 5053) and sterilized water. Diet treatments (Table 1) were initiated at 6-weeks of age (*n* = 12 per diet and sex), with cages randomly assigned by E.B.P.L. to Chow (14.7% kcal fat; #5L79, Charles River Laboratories; nutritionally similar to NIH31M) or HFD (60% kcal fat; #D12492i, Research Diets) within each cohort. Diets were provided *ad libitum* until the end of the study. Body mass was measured weekly, and body composition (i.e., lean and fat mass) was measured by quantitative magnetic resonance (EchoMRI) at 5 and 25 weeks of age. Energy expenditure and the respiratory exchange ratio (RER) were also measured at 5 and 25 weeks of age by indirect calorimetry using a multiple animal respirometry system (Sable Systems, USA), as previously described [18]. 10-min/animal averages were collected hourly over a continuous 20-h period, and the average RER values were reported for the light and dark phases to evaluate sex-dependent effects of HFD on carbohydrate versus fat oxidation. Glucose tolerance testing was performed at 25 weeks of age as previously described [13]. At 26 weeks of age, animals were moved to the lab in transport cages for a period of 1–2 h prior to blood collection between 9-11AM. Blood was collected as a terminal procedure by cardiac puncture under isoflurane anesthesia, and serum was extracted and stored in aliquots at -80 °C until analysis, as previously described [13]. Tissues were collected immediately following euthanasia.

### Serum analysis

Serum metabolites were measured in a subset of samples using two different methods following manufacturer instructions: (1) IDEXX Catalyst Dx Chemistry Analyzer (USA) with Chem 17 and Triglycerides panels, and (2) gas-chromatography mass-spectrometry (GC-MS). IDEXX analysis included *n* = 8 samples per group, with sample selection prioritized for animals with the greatest number of proteins detected in the targeted cartilage proteomic analysis. Within these samples, *n* = 4 per group were selected for GC-MS analysis based on serum availability. Metabolites were extracted, derivatized, and analyzed by GC-MS (Agilent 7890B-5977 A), as previously described [19], with ribitol used as an internal standard for normalization.

### Microbiome analysis

Cecal material was obtained following death and immediately frozen in liquid nitrogen. Microbial DNA was extracted using a QIAamp DNA microbiome kit (Qiagen). A 460 bp amplicon from the V3-V4 region of the bacterial 16 S rRNA gene was amplified using a high-fidelity polymerase (NEB Q5, New England Biolabs), and deep sequencing was performed at the OMRF Clinical Genomics Center on an Illumina miSeq sequencer using a 300 bp paired-end sequencing protocol, as previously described [20]. Microbial analyses were performed using the Quantitative Insights into Microbial Ecology (QIIME2) software package, amplicon analysis v2023.9 [21]. Demultiplexed raw sequences were quality filtered, denoised, and chimeras removed using deblur [22]. Taxonomy was assigned to amplicon sequence variants via a custom Naïve Bayes taxonomic classifier trained on the GreenGenes2 99% OTUs full-length sequence dataset. Eight samples with low microbial feature reads (< 18,000/sample) were excluded, leaving 37 samples for analysis (6 chow female, 9 chow male, 10 HFD female, and 12 HFD male). Data were then rarefied to 18,284 reads/sample. Alpha diversity was calculated using a phylogenetic index (Faith’s Phylogenetic Diversity), with group differences evaluated by Student’s t-test (normal distribution confirmed Kolmogorov-Smirnov test, all *p* > 0.05). Beta diversity was characterized using an unweighted UniFrac model. Group differences were calculated using a permuted analysis of variance (PERMANOVA) test with 999 permutations using a pseudo-F cluster variance approach, with significance evaluated by Kruskal-Wallis test using Benjamini-Hochberg multiple comparison correction [20]. Group microbiome composition differences were evaluated by linear discriminant analysis effect size (LEfSe) pipeline, with an LDA effect size ≥ 2.0 set as the threshold for significance [23].

### Joint structural analysis

The left knee joint was processed for semi-quantitative OA histopathology grading, as previously described [24]. Briefly, 2–3 coronal sections from the mid-joint load-bearing region were stained with hematoxylin, Fast Green, and Safranin-O for blinded grading of cartilage pathology, which was performed separately for the medial tibia, medial femur, lateral tibia, and lateral femur using OARSI scoring (0–6) and averaged. Osteophyte pathology was evaluated in the medial tibia (0–3). Synovitis scoring was performed adjacent to the tibia in the medial and lateral compartments based on the presence (score = 1) or absence (score = 0) of synovial lining cell hyperplasia (≥ 3 cell layers). Slides were organized by knee joint sample, randomized by diet and sex, and assigned a temporary identification code by D.C. to blind graders (T.M.G. and P.M.D.) and minimize any order effect. Scores that differed by > 1 between graders were re-evaluated for consensus scoring. Scores were then averaged for both graders to obtain a final score per section and location. Final scores represent the overall average score per animal for each category (e.g., cartilage, synovium, osteophyte).

### Targeted gene expression and proteomic analyses

Immediately following death, we harvested gonadal adipose tissue, the infrapatellar fat pad with adjacent synovium (IFP-synovium), and the articular cartilage from the right knee for RNA and protein isolation, as previously described [11, 13]. Briefly, tissues were frozen at minus 80 °C in TRIzol™ (Invitrogen, USA) and then processed with RNA Clean and Concentrator Columns (Zymo Research, USA) per manufacturer’s protocol. Cartilage protein in the lower organic phase fraction from the TRIzol™ extraction was further processed as previously described for mass spectrometry [13, 25]. Gonadal adipose tissue pro-inflammatory gene expression was quantified by qRT-PCR, as previously described [11], for *Lep* [FWD: AGCGAGGAAAATGTGCTGGA, REV: TGAAGCCCGGGAATGAAGTC], *Ccl2* [FWD: AGGTCCCTGTCATGCTTCTGG, REV: CTGCTGCTGGTGATCCTCTTG], and *Tnf* [FWD: AATGGCCTCCCTCTCATCAGTT, REV: CCACTTGGTGGTTTGCTACGA]. Delta Ct values were calculated relative to the geomean of *Hprt* [FWD: AAGCTTGCTGGTGAAAAGGA, REV: TCCACTTTCGCTGATGACAC] and *Hsp90ab* [FWD: CGGGAAAGAGCTGAAGATTG, REV: GCAGAGTAGAAGCCGACACC]. IFP-synovium and cartilage gene expression were quantified using targeted Fluidigm DELTAgene Assays (Supplemental Tables S1 and S2) conducted following manufacturer instructions using a Fluidigm 96.96 Dynamic Array IFC and Biomark HD instrument [24]. RStudio with stats package was used to compute delta-Ct values of target genes by subtracting the geometric mean of Ct values of 5 reference genes (IFP-synovium: *Actb*,* B2m*,* Gapdh*,* Hprt*,* Rpl14*; cartilage: *Actb*,* B2m*,* Gapdh*,* Gusb*,* Hsp90ab1*). The relative abundance of 101 proteins involved in cellular metabolism and redox homeostasis were quantified using mass spectrometry selective reaction monitoring (SRM), as previously reported [13, 25]. The abundance of each target protein was calculated using the geomean of two peptide areas normalized to the area of a stable reference protein, Hspd1.

### Chondrocyte metabolic profiling

In vivo chondrocyte metabolism was evaluated in a separate cohort of 5-week-old male and female C57BL/6J mice fed a chow diet (LabDiet 5053). Femoral head (hip) cartilage was isolated from animals immediately following death and prepared for GC-MS semi-targeted metabolic profiling as previously described [26]. Cartilage was pooled from 4 animals per biological replicate, and 4–5 biological replicates were measured for each sex. Metabolite relative abundance was calculated by peak area normalized to sample wet weight and internal standard (ribitol) [26]. Sex differences in in vitro chondrocyte metabolism were evaluated as previously described using a Seahorse XFe24 Analyzer (Agilent) in passage one primary immature chondrocytes isolated from knee epiphyseal cartilage of 6 to 8-day old mice [24]. Briefly, 3 × 10^5^ cells were seeded in 12-well plates in 1 ml DMEM medium (low glucose [1000 mg/L], pyruvate [110 mg/L], GlutaMAX™; Life Technology, 10567014) supplemented with 10% fetal bovine serum (FBS) and 1% penicillin/streptomycin (P/S) and cultured in a humidified incubator at 37ºC under 5% CO_2_ and ambient oxygen. Cells were expanded to approximately 90% confluency and then released and seeded in 24-well Seahorse microplates at 60,000 cells per well one day before testing. Cells were also seeded in parallel Seahorse microplates for cell counting and Seahorse data normalization. Glycolytic Rate and Mitochondrial Stress assays were performed following manufacturer instructions, with independent biological samples tested in duplicate on the same plate and averaged. Cells were harvested from 9 to 12 C57BL/6J mice per sex, which were littermates from 4 litters.

### Statistical analyses

Animal experiment sample sizes were based on power analyses for OA histopathology, our primary outcome. Using data from prior HFD studies in our lab, *n* = 9 animals per group was estimated to provide 80% power to detect a 30% difference in mean OARSI scores with a significance level of *p* = 0.05 (mean OARSI score of 1.0 and standard deviation of 0.2). We tested *n* = 12 per group in case of unexpected animal health or tissue processing issues. No animals died prior to study completion, although some outcomes included *n* < 12 per group due to technical issues with tissue processing, insufficient sample availability, or assay limitations, as summarized in Supplemental Table S3. Sex and diet effects were evaluated by two-way ANOVA. Data that did not meet test assumptions for normality or homoscedasticity, even after log transformation, were analyzed by Kruskal-Wallis or Wilcoxon tests. Tests showing a significant effect of diet, sex, or an interaction (*p* < 0.05), were followed up with multiple-comparison post-hoc tests to identify individual group differences as specified in figure legends. Differential gene expression analyses were based on 2-tailed Student’s t-Test of log2 transformed data as defined in figure legends. Hierarchical clustering analyses were performed to identify genes and proteins that shared similar expression patterns in response to sex or diet comparisons. Gene and protein data were standardized by subtracting the mean and dividing by the standard deviation, and Ward’s minimum variance method was used to calculate cluster distances. Additional statistical details are provided in figure legends.

## Results

### Systemic metabolic response to HFD

**Fig. 1:**
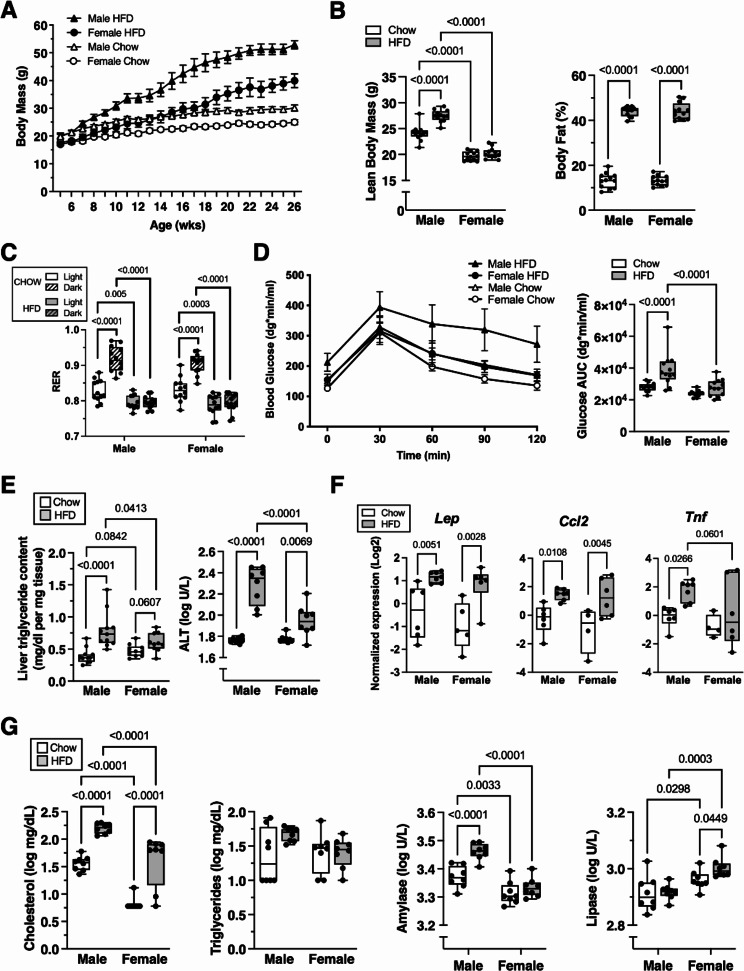
Systemic metabolic phenotyping of HFD effects in male and female mice. **A** Time course of body mass change in male and female mice fed Chow or HFD from 6 to 26 weeks of age. (Three-way RM-ANOVA: Sex [*p* < 0.0001], Diet [*p* < 0.0001], Time [*p* < 0.0001]). **B** Lean body mass and body fat percentage measured by QMR at 25 weeks of age. (Lean body mass two-way ANOVA: Sex [*p* < 0.0001], Diet [*p* < 0.0001], Interaction [*p* = 0.0001]; Body fat two-way ANOVA: Sex [*p* = 0.96], Diet [*p* < 0.0001], Interaction [*p* = 0.89]). **C** Respiratory exchange ratio (RER) measured by indirect calorimetry at 25 weeks of age. (Three-way ANOVA: Sex [*p* = 0.52], Diet [*p* < 0.0001], Circadian [*p* < 0.0001], Diet x Circadian [*p* < 0.0001]). **D** Blood glucose concentration time course and area under the curve (AUC) obtained from glucose tolerance test measured at 25 weeks of age. (Glucose time course three-way RM-ANOVA: Sex [*p* = 0.0253], Diet [*p* = 0.0216], Time [*p* < 0.0001]; AUC two-way ANOVA: Sex [*p* < 0.0001], Diet [*p* = 0.0002], Interaction [*p* = 0.0434]). **E** Liver triglyceride content and serum alanine transaminase (ALT), endpoint measurements (Liver triglycerides two-way ANOVA: Sex [*p* = 0.81], Diet [*p* < 0.0001], Interaction [*p* = 0.0091]; ALT two-way ANOVA: Sex [*p* = 0.0006], Diet [*p* < 0.0001], Interaction [*p* = 0.0003]). **F** Gonadal adipose tissue gene expression, endpoint measurements (*Lep* two-way ANOVA: Sex [*p* = 0.21], Diet [*p* = 0.0002], Interaction [*p* = 0.71]; *Ccl2* two-way ANOVA: Sex [*p* = 0.33], Diet [*p* = 0.0004], Interaction [*p* = 0.56]; *Tnf* two-way ANOVA: Sex [*p* = 0.079], Diet [*p* = 0.028], Interaction [*p* = 0.46]). **G** Serum metabolites, endpoint measurements (Cholesterol two-way ANOVA: Sex [*p* < 0.0001], Diet [*p* < 0.0001], Interaction [*p* = 0.41]; Triglycerides two-way ANOVA: Sex [*p* = 0.28], Diet [*p* = 0.11], Interaction [*p* = 0.10]; Amylase two-way ANOVA: Sex [*p* < 0.0001], Diet [*p* = 0.0001], Interaction [*p* = 0.0086]; Lipase two-way ANOVA: Sex [*p* < 0.0001], Diet [*p* = 0.11], Interaction [*p* = 0.20]). Values are mean ± 95%CI. For boxplots, boxes represent the 25th to 75th percentiles, horizontal line indicates the median, and whiskers demonstrate maximum and minimum values. Fisher’s LSD post-hoc paired comparisons shown for *p* < 0.10. Sample sizes for each analysis provided in Supplemental Table S3

After 20-weeks of diet treatment, body weights differed across each sex and diet group, being least in female Chow and greatest in male HFD animals (Fig. 1A). As expected, HFD caused substantially greater weight gain compared to Chow during the 20-week treatment (2.37-fold weight increase versus 1.39-fold; mean difference [95%CI] = 0.98-fold [0.87–1.09]). The relative weight gain within Chow animals was similar in both sexes (mean difference [95%CI] = -0.04-fold [-0.15–0.07], female vs. male), whereas the response to HFD was slightly reduced in females (-0.25-fold [-0.50–0.01]). Notably, lean body mass increased with HFD treatment versus Chow in males but not females (Fig. 1B), and percent body fat increased with HFD to a similar degree in both sexes (Fig. 1B). We measured RER to assess the oxidation of carbohydrates (value = 1.0) versus fats (value = 0.7), and in both sexes, HFD treatment reduced RER values during the light and dark phases and suppressed circadian fluctuations (Fig. 1C). Impaired glucose homeostasis was evaluated by glucose tolerance testing. Fasting glucose and post-challenge area under the curve (AUC) glucose levels were elevated in male HFD but not female HFD mice (Fig. 1D). Hepatic health was evaluated by liver triglyceride content and serum alanine transaminase (ALT). HFD increased liver triglycerides and ALT levels in males and females versus Chow groups, although the relative and absolute increases in both biomarkers were greater in males versus females (Fig. 1E). Gonadal adipose inflammation was assessed by quantitative gene expression of the adipokine leptin (*Lep*) and the chemokine *Ccl2*, which both increased > 2-fold with HFD in males and females (Fig. 1F). *Tnf* expression also increased with HFD in males but not females (Fig. 1F). Numerous sex and diet differences were also observed in serum metabolites. HFD increased serum cholesterol in both males and females, with greater overall levels in males (Fig. 1G). HFD increased serum amylase in males only, and amylase levels were greater overall in males (Fig. 1G). In contrast, HFD increased lipase levels in females only, even as overall lipase levels were greater in females.

**Fig. 2:**
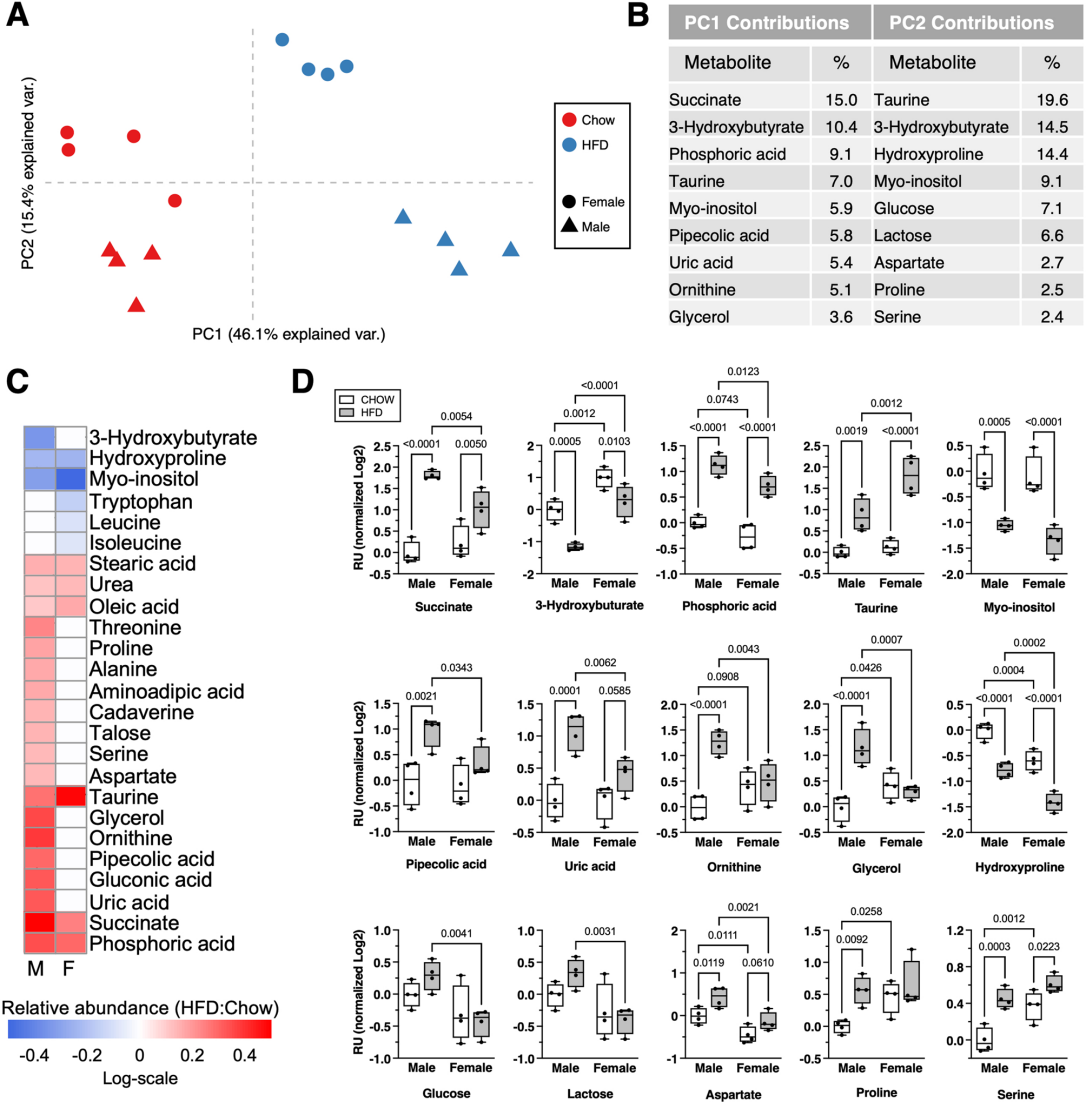
Effect of HFD on serum metabolites in male and female mice. **A** Principal component analysis based on relative abundance values of 49 serum metabolites measured by GC-MS. Symbols represent individual samples (*n* = 4 per group) according to diet treatment (Chow = red, HFD = blue) and biological sex (female = circle, male = triangle). Principal component 1 (PC1) primarily differentiates between diet treatments, while PC2 primarily differentiates between sexes. **B** Descending list of the top nine metabolites with the greatest partial contributions to the percentage of variance contributed to PC1 and PC2. **C** Heatmap of metabolites significantly altered by HFD, expressed as log-scale relative abundance. Diet effects were initially screened by two-way ANOVA, and metabolites showing significance (*p* < 0.05) were subsequently evaluated by t-test in a sex-specific manner. Significant effects of HFD (*p* < 0.05) are indicated by the presence of a heatmap color, whereas white indicates *p* ≥ 0.05. **D** Serum metabolite values for metabolites shown in panel B, expressed as relative units normalized to male Chow average and log2 transformed. Boxplots represent the 25th to 75th percentiles, horizontal line indicates the median, and whiskers demonstrate maximum and minimum values. Fisher’s LSD post-hoc paired comparisons shown for *p* < 0.10. Two-way ANOVA values: Succinate (Sex [*p* = 0.125], Diet [*p* < 0.0001], Interaction [*p* = 0.0086]); 3-Hydroxybutyrate (Sex [*p* < 0.0001], Diet [*p* = 0.0001], Interaction [*p* = 0.24]); Phosphoric acid (Sex [*p* = 0.0047], Diet [*p* < 0.0001], Interaction [*p* = 0.50]); Taurine (Sex [*p* = 0.0053], Diet [*p* < 0.0001], Interaction [*p* = 0.024]); Myo-inositol (Sex [*p* = 0.24], Diet [*p* < 0.0001], Interaction [*p* = 0.48]); Pipecolic acid (Sex [*p* = 0.087], Diet [*p* = 0.0018], Interaction [*p* = 0.16]); Uric acid (Sex [*p* = 0.0417], Diet [*p* = 0.0002], Interaction [*p* = 0.0332]); Ornithine (Sex [*p* = 0.26], Diet [*p* = 0.0009], Interaction [*p* = 0.0026]); Glycerol (Sex [*p* = 0.14], Diet [*p* = 0.0021], Interaction [*p* = 0.0004]); Hydroxyproline (Sex [*p* < 0.0001], Diet [*p* < 0.0001], Interaction [*p* = 0.83]); Glucose (Sex [*p* = 0.0041], Diet [*p* = 0.58], Interaction [*p* = 0.17]); Lactose (Sex [*p* = 0.004], Diet [*p* = 0.46], Interaction [*p* = 0.12]); Aspartate (Sex [*p* = 0.0004], Diet [*p* = 0.004], Interaction [*p* = 0.54]); Proline (Sex [*p* = 0.06], Diet [*p* = 0.0143], Interaction [*p* = 0.15]); Serine (Sex [*p* = 0.0012], Diet [*p* = 0.0002], Interaction [*p* = 0.11])

Semi-targeted GC-MS analysis of serum metabolites revealed additional diet and sex effects, as indicated by principal component (PC) analysis (Fig. 2A). Variance along PC1 primarily corresponded to diet effects, whereas PC2 primarily indicated sex differences. The top metabolic contributors to PC1 and PC2 were mostly distinct (Fig. 2B), although some metabolites contributed to both PCs due to sex differences in relative abundance or magnitude of change with HFD (e.g., 3-hydroxybutyrate, taurine, and myo-inositol) (Fig. 2C, D). Overall, HFD altered the relative abundance of 25/49 serum metabolites, with most differences observed in males (22/49) and half as many occurring in females (11/49) (Fig. 2C). Pathway analysis identified sexually dimorphic responses to HFD, such as changes in aminoacyl-tRNA biosynthesis and arginine biosynthesis in males and branched-chain amino acid metabolism in females (Supplemental Figure S1).

### Sexually dimorphic changes in gut microbiome to HFD

**Fig. 3:**
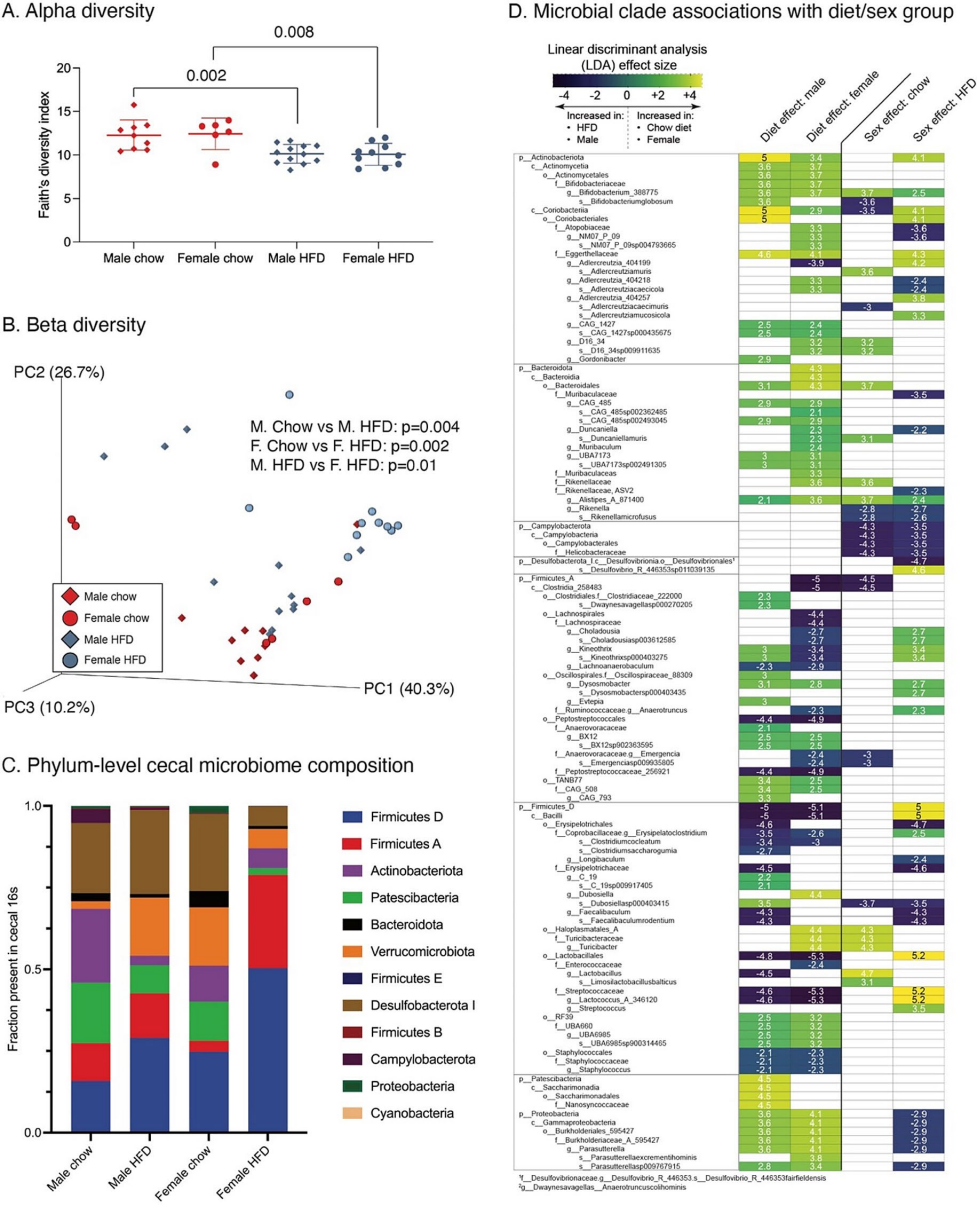
Effect of HFD on cecal microbial 16 S metagenomic diversity and compositional analyses in male and female mice. Microbial analyses were performed using the Quantitative Insights into Microbial Ecology (QIIME2) software package. **A** HFD reduced the alpha diversity index in both male and female mice. Values are mean ± SD (p-values shown for *p* < 0.10 based on Student’s t-tests). **B** Effect of diet and sex on microbial beta diversity characterized using an unweighted UniFrac model. Group differences evaluated using a permuted analysis of variance (PERMANOVA) test, with significance evaluated by Kruskal-Wallis test using Benjamini-Hochberg multiple comparison correction. **C** Stacked bar chart comparing the average phylum-level cecal microbiome fractional composition within each experimental group. **D** Group 16 S microbiome composition differences evaluated by linear discriminant analysis (LDA) effect size; only statistically significant clade differences are presented based on LDA threshold of ≥ 2 or ≤-2, corresponding to *p* ≤ 0.01. Comparisons organized by diet effects within each sex and sex effects within each diet. Numbers and heatmap color scheme represent effect sizes, with negative values corresponding to increased clade abundance in male mice and HFD and positive values corresponding to increased clade abundance in female mice and Chow diet

We first analyzed alpha diversity using Faith’s diversity index. Alpha diversity was significantly lower with HFD versus Chow diet in both sexes (Fig. 3A). However, we did not observe significant sex differences in alpha diversity in either diet group (Chow: female vs. male, *p* = 0.9; HFD: female vs. male, *p* = 0.9). Turning to beta diversity, we found significant differences with HFD versus Chow in both male (*p* = 0.004) and female (*p* = 0.002) mice. We also observed sex differences in beta diversity among HFD fed animals (*p* = 0.001), but not among Chow fed animals (*p* = 0.3) (Fig. 3B). When comparing Chow versus HFD, we identified 67 clade differences in male mice (Supplementary Table S4a) and 76 clade differences in female mice (Supplementary Table S4b) (Fig. 3C and D). Many of these clades (44) were altered by diet in both male and female animals. Of these 44, the vast majority (42) were of concordant directionality (i.e., enriched or depleted with Chow or HFD) in both sexes (Supplementary Table S4c). Next, comparing microbiome changes by sex, we observed 27 clade differences in Chow fed mice and 50 clade differences in HFD fed mice (Supplementary Tables S4d and e). Of these, 11 clades were shared between diets, and 9 of these shared clades were concordantly enriched or depleted (Supplementary Table S4f). Among those clades decreased with HFD in both sexes were members of phylum *Actinobacteria*, phylum *Bacteroidota*, and phylum *Proteobacteria*, particularly class *Gammaproteobacteria*. Conversely, family *Streptococcaceae*, phylum *Firmicutes*, and family *Peptostreptococcaceae*, among others, were increased with HFD in mice of both sexes. Several effects, though, were diet or sex dependent. For example, within *Bacteroidota*, genus *Alistipes* was more abundant in female mice versus male mice fed Chow (40–fold higher in F than M, *P* = 7.7E-6), and this sex difference became greater with HFD (51–fold higher in F than M, *P* = 0.02). In addition, two microbiome clades differed between sexes only under HFD conditions. Specifically, the genus *Lactococcus* was 102–fold more abundant in female versus male mice (*p* = 1.3E-7), and *Streptococcus* was 3.5–fold more abundant in female versus male mice (*p* = 0.02) (Fig. 3D).

### Effect of sex and HFD on IFP-synovium gene expression

**Fig. 4:**
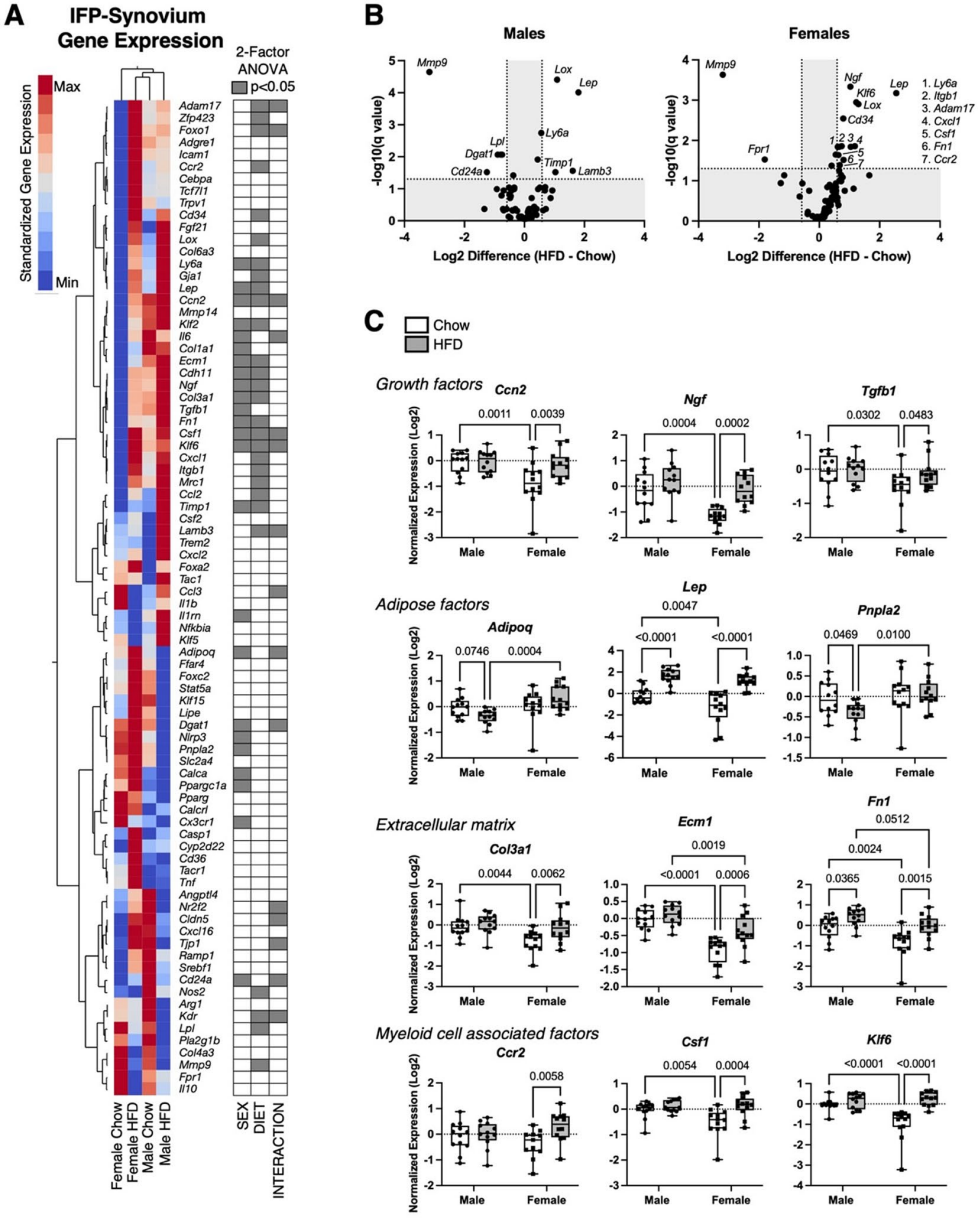
Effect of HFD on IFP-synovium gene expression in male and female mice. **A** 2-way hierarchical clustering analysis of IFP-synovium gene expression values versus experimental groups. Heatmap color legend signifies standardized expression values calculated by subtracting the mean and dividing by the standard deviation. Two-factor ANOVA chart indicates significant factor effects for genes in corresponding rows. **B** Sex-specific effects of HFD on gene expression evaluated by log2 transformed volcano plot comparisons. Labelled genes have false discovery rate adjusted (Q = 5%) significance (q < 0.05) and minimum log 2-transformed differential expression value ±0.585 (1.5-fold change). **C** Expression data for selected genes presented as log 2-transformed fold-change normalized to male Chow diet samples using the delta-delta Ct method. Boxplots represent the 25th to 75th percentiles, horizontal line indicates the median, and whiskers demonstrate maximum and minimum values. Fisher’s LSD post-hoc paired comparisons shown for *p* < 0.10. Two-way ANOVA values: *Ccn2* (Sex [*p* = 0.0064], Diet [*p* = 0.032], Interaction [*p* = 0.042]); *Ngf* (Sex [*p* = 0.0006], Diet [*p* = 0.0002], Interaction [*p* = 0.087]); *Tgfb1* (Sex [*p* = 0.048], Diet [*p* = 0.089], Interaction [*p* = 0.26]); *Adipoq* (Sex [*p* = 0.0047], Diet [*p* = 0.89], Interaction [*p* = 0.018]); *Lep* (Sex [*p* = 0.0059], Diet [*p* < 0.0001], Interaction [*p* = 0.20]); *Pnpla2* (Sex [*p* = 0.025], Diet [*p* = 0.17], Interaction [*p* = 0.14]); *Col3a1* (Sex [*p* = 0.0087], Diet [*p* = 0.014], Interaction [*p* = 0.14]); *Ecm1* (Sex [*p* < 0.0001], Diet [*p* = 0.0021], Interaction [*p* = 0.057]); *Fn1* (Sex [*p* = 0.0006], Diet [*p* = 0.0003], Interaction [*p* = 0.39]); *Ccr2* (Sex [*p* = 0.94], Diet [*p* = 0.022], Interaction [*p* = 0.082]); *Csf1* (Sex [*p* = 0.047], Diet [*p* = 0.0015], Interaction [*p* = 0.042]); *Klf6* (Sex [*p* = 0.0042], Diet [*p* < 0.0001], Interaction [*p* = 0.0007]). Sample sizes for each analysis provided in Supplemental Table S3

We evaluated the effects of sex and HFD on the joint microenvironment by quantifying transcript levels of 82 genes involved in regulating inflammation, adipose tissue homeostasis, and fibrosis in IFP-synovium tissue (Fig. 4A). Overall, sex and diet altered the expression of over half the genes (44) (*p* < 0.05), with many of these genes (24) altered by both sex and HFD or a sex-diet interaction. Changes observed in both males and females included HFD-induced upregulation of *Lep*, *Lox*, and *Ly6a* and downregulation of *Mmp9* (Fig. 4B). Compared to males, females expressed lower levels of extracellular matrix associated genes *Col1a1*, *Ecm1*, and *Fn1*. Most notable, though, were the sex-dependent differences in response to HFD. Volcano plots revealed a symmetrical up/down regulation of gene expression changes with HFD versus Chow in males, whereas females were biased towards upregulation (Fig. 4B). For example, only in females did HFD upregulate (*p* < 0.05) the expression of numerous genes involved in fibrosis and inflammation (e.g., *Col3a1*,* Ecm1*,* Fn1*,* Ccr2*,* Csf1*,* Klf6*,* Cxcl1*,* Itgb1*, and *Adam17*; Fig. 4B, C). Thus, while most systemic metabolic effects of HFD occurred in males, local effects of HFD on fibrosis and inflammation gene transcripts in IFP-synovium tissue were mainly observed in females.

### Sexually dimorphic effect of HFD on knee OA histopathology

**Fig. 5:**
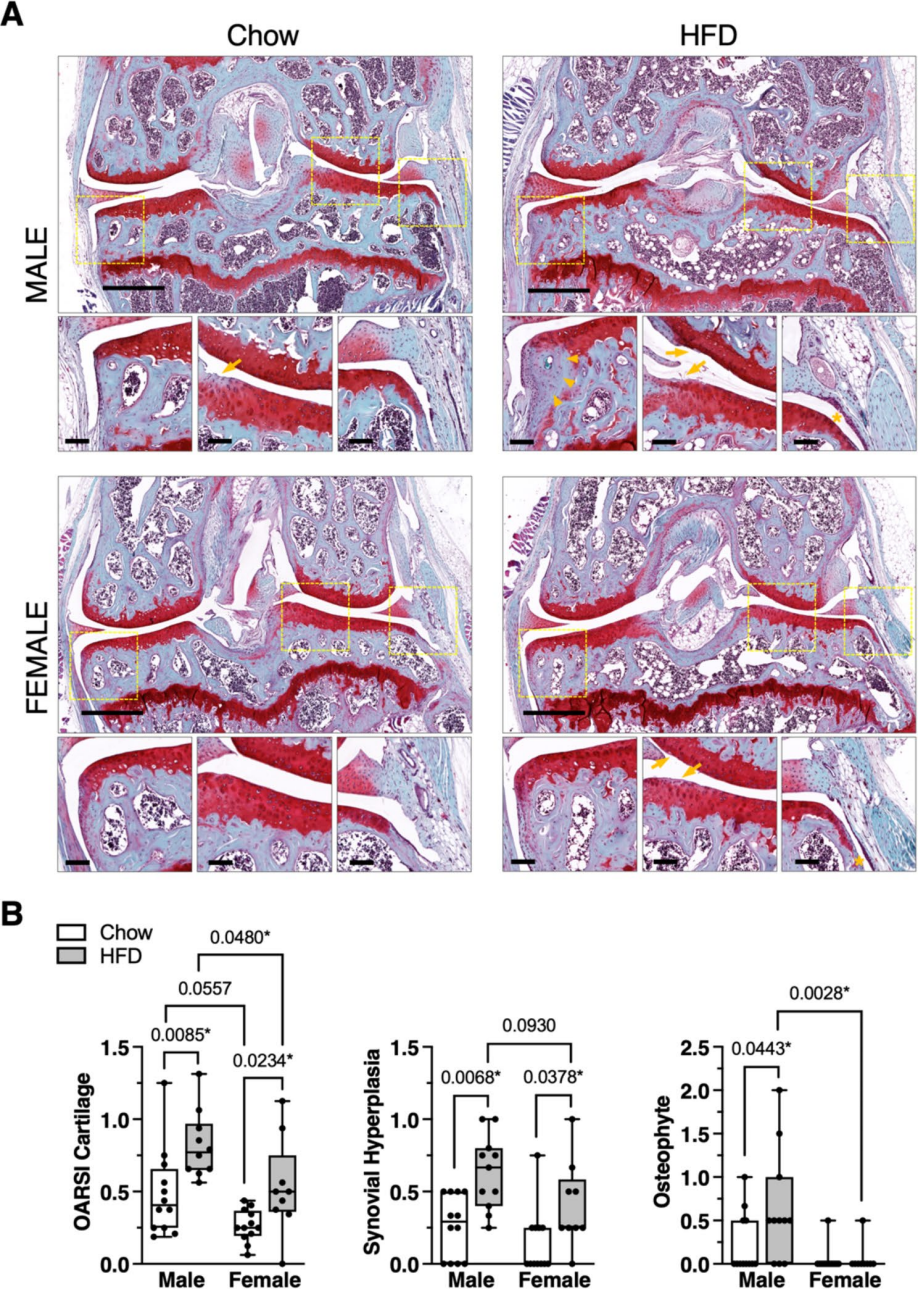
Male and female mice develop greater knee OA pathology with HFD versus chow. **A** Representative histological images of mid-coronal knee sections stained with Safranin-O, fast green, and hematoxylin from male and female mice fed Chow or HFD. Large panel scale bar = 500 μm. Dashed boxes in large panel indicate locations selected for small panel images representing, from left to right, the medial joint margin, lateral femoral-tibial cartilage loading region, and synovial lining inferior to the lateral meniscus. In small panels, arrowheads indicate osteophyte development (male HFD), arrows indicate mild cartilage damage (male Chow, male HFD, female HFD), and asterisks indicate synovial thickening (male HFD, female HFD). Small panel scale bars = 100 μm. **B** Semi-quantitative histological grading of cartilage, synovium, and osteophyte pathology. All scoring was performed by 2 experienced graders in a blinded manner. OARSI cartilage score range = 0–6; Synovial hyperplasia score range = 0 (absent) or 1 (present); Osteophyte score range = 0–3. Data points represent average values for individual animals. Boxes represent the 25th to 75th percentiles, horizontal line indicates the median, and whiskers demonstrate maximum and minimum values. Data were analyzed by Kruskal-Wallis test: OARSI cartilage (*p* = 0.0002), Synovial hyperplasia (*p* = 0.0008), Osteophyte (*p* = 0.0035). Post-hoc paired comparisons based on two-stage linear step-up procedure of Benjamini, Krieger and Yekutieli to control for 10% false discovery rate, q (*p* < 0.10 shown; *q < 0.10). Sample sizes for each analysis provided in Supplemental Table S3

20 weeks of HFD induced mild cartilage pathology characterized by focal reduction in Safranin-O staining, small surface fibrillations, and occasional clefts below the superficial layer with loss of surface lamina (Fig. 5A). Cartilage pathology was greater in the lateral versus medial compartment articular surfaces under both diet conditions (mean difference in OARSI score [95% CI]; Chow: 0.48 [0.35–0.62]; HFD: 0.60 [0.43–0.78]). HFD caused greater cartilage pathology versus Chow in both males (*p* = 0.0085) and females (*p* = 0.0234), with overall pathology being higher in males versus females under HFD conditions (*p* = 0.0480) (Fig. 5B). HFD also increased synovial hyperplasia in males (*p* = 0.0068) and females (*p* = 0.0378) compared to Chow conditions (Fig. 5B). However, osteophytes developed in a sexually dimorphic manner in response to HFD, forming in male (*p* = 0.0443) but not female mice (Fig. 5B).

### Effect of sex and HFD on cartilage gene expression and protein abundance

**Fig. 6:**
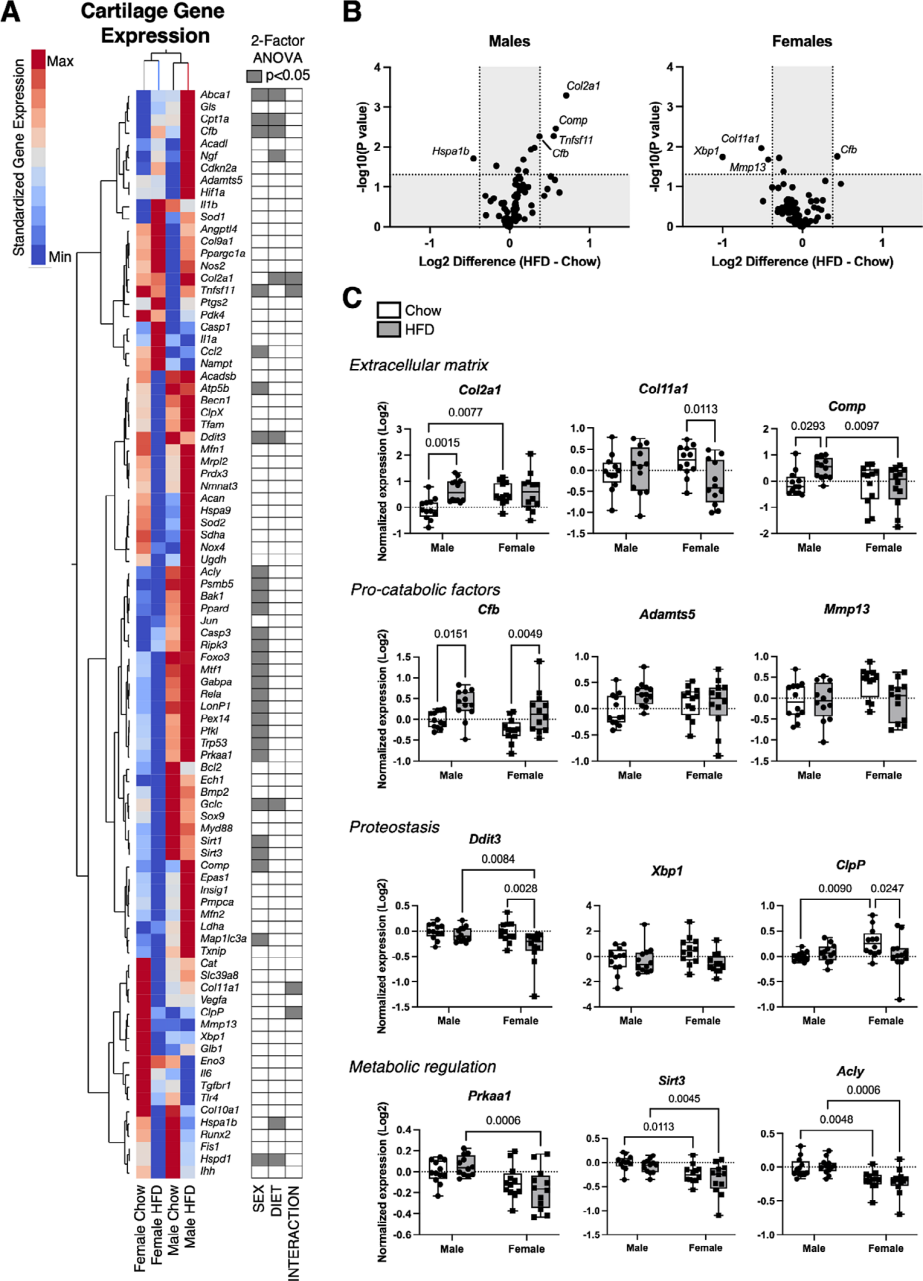
Effect of HFD on cartilage gene expression in male and female mice. **A** 2-way hierarchical clustering analysis of cartilage gene expression values versus experimental groups. Heatmap color legend signifies standardized expression values calculated by subtracting the mean and dividing by the standard deviation. Two-factor ANOVA chart indicates significant factor effects for genes in corresponding rows. **B** Sex-specific effects of HFD on gene expression evaluated by log2 transformed volcano plot comparisons. Labelled genes have *p* < 0.05 and minimum log 2-transformed differential expression value ±0.378 (1.3-fold change). Note the analysis does not include a false discovery rate correction and should be considered exploratory. **C** Expression data for selected genes presented as log 2-transformed fold-change normalized to male Chow diet samples using the delta-delta Ct method. Boxplots represent the 25th to 75th percentiles, horizontal line indicates the median, and whiskers demonstrate maximum and minimum values. Fisher’s LSD post-hoc paired comparisons shown for *p* < 0.10. Two-way ANOVA values: *Col2a1* (Sex [*p* = 0.091], Diet [*p* = 0.013], Interaction [*p* = 0.032]); *Col11a1* (Sex [*p* = 0.89], Diet [*p* = 0.12], Interaction [*p* = 0.038]); *Comp* (Sex [*p* = 0.049], Diet [*p* = 0.17], Interaction [*p* = 0.079]); *Cfb* (Sex [*p* = 0.047], Diet [*p* = 0.0003], Interaction [*p* = 0.76]); *Adamts5* (Sex [*p* = 0.88], Diet [*p* = 0.15], Interaction [*p* = 0.10]); *Mmp13* (Sex [*p* = 0.083], Diet [*p* = 0.083], Interaction [*p* = 0.11]); *Ddit3* (Sex [*p* = 0.041], Diet [*p* = 0.010], Interaction [*p* = 0.079]); *Xbp1* (Sex [*p* = 0.38], Diet [*p* = 0.084], Interaction [*p* = 0.11]); *ClpP* (Sex [*p* = 0.082], Diet [*p* = 0.24], Interaction [*p* = 0.043]); *Prkaa1* (Sex [*p* = 0.0005], Diet [*p* = 0.92], Interaction [*p* = 0.14]); *Sirt3* (Sex [*p* = 0.0002], Diet [*p* = 0.17], Interaction [*p* = 0.81]); *Acly* (Sex [*p* < 0.0001], Diet [*p* = 0.96], Interaction [*p* = 0.60]). Sample sizes for each analysis provided in Supplemental Table S3

We evaluated the effects of sex and HFD on cartilage by quantifying transcript levels of 89 genes involved in regulating extracellular matrix homeostasis, inflammation, metabolism, and cellular stress (Fig. 6A). Overall, sex or diet altered the expression of about one-third of the genes (33), with a subset of these genes (10) altered by both sex and HFD or a sex-diet interaction. Most differences in gene expression were sex-dependent (28), with many metabolic and cellular stress genes more highly expressed in males versus females (e.g., *Acly*, *Psmb5*, *Bak1*, *Ppard*, *Casp3*, *Ripk3*, *Trp53*, *Prkaa1*, *Sirt1*, and *Sirt3*) (Fig. 6A, C). Complement factor b (*Cfb*) was the only gene upregulated by a HFD in males (*p* = 0.0151) and females (*p* = 0.0049), whereas no genes were uniformly downregulated by HFD in both sexes (Fig. 6B). Many transcriptional changes were sexually dimorphic, with HFD upregulating the expression of *Col2a1*, *Comp*, and *Tnfsf11* in males only (Fig. 6B, C). In contrast, HFD downregulated the expression of *Col11a1*, *Ddit3*, and *ClpP* in females only (Fig. 6B, C).

**Fig. 7:**
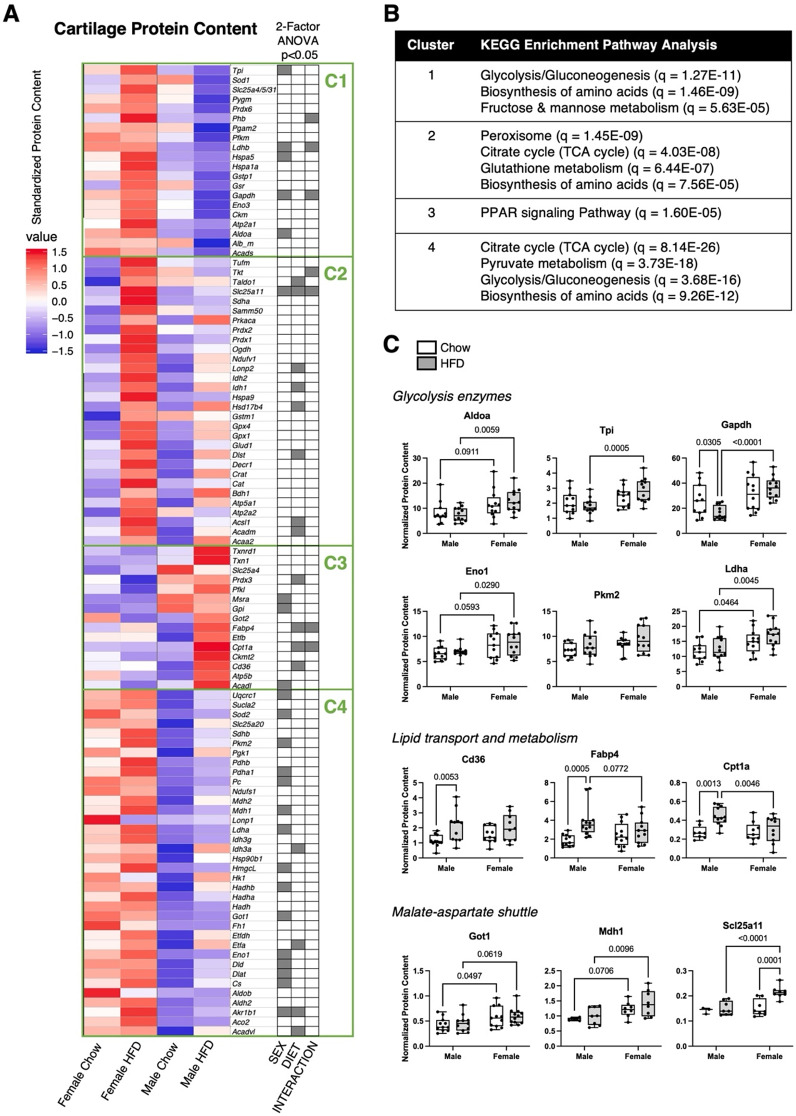
Effect of HFD on cartilage protein content in male and female mice. Protein abundance was measured by selected reaction monitoring (SRM) mass spectrometry in cartilage harvested from knees of male and female mice fed Chow or HFD from 6 to 26 weeks of age. **A** Heat map of log-transformed protein abundance values normalized to the median, across both diet conditions and sexes. Proteins were grouped in clusters based on Euclidian distance dissimilarity matrix and partitioning around mediods algorithm. Cluster numbers (C1-C4) determined by silhouette algorithm. Two-factor ANOVA chart indicates significant factor effects for proteins in corresponding rows. **B** Top significant predicted pathways (qFDR < 0.05) associated with proteins in each cluster based on pathway analysis performed using STRING database over KEGG background. **C** Protein abundance data for selected proteins normalized to sample’s total ion current. Boxplots represent the 25th to 75th percentiles, horizontal line indicates the median, and whiskers demonstrate maximum and minimum values. Fisher’s LSD post-hoc paired comparisons shown for *p* < 0.10. Two-way ANOVA values: Aldoa (Sex [*p* = 0.0022], Diet [*p* = 0.91], Interaction [*p* = 0.43]); Tpi (Sex [*p* = 0.0010], Diet [*p* = 0.70], Interaction [*p* = 0.095]); Gapdh (Sex [*p* = 0.0002], Diet [*p* = 0.38], Interaction [*p* = 0.026]); Eno1 (Sex [*p* = 0.0050], Diet [*p* = 0.47], Interaction [*p* = 0.84]); Pkm2 (Sex [*p* = 0.045], Diet [*p* = 0.092], Interaction [*p* = 0.82]); Ldha (Sex [*p* = 0.0009], Diet [*p* = 0.21], Interaction [*p* = 0.53]); Cd36 (Sex [*p* = 0.53], Diet [*p* = 0.0043], Interaction [*p* = 0.28]); Fabp4 (Sex [*p* = 0.64], Diet [*p* = 0.0021], Interaction [*p* = 0.042]); Cpt1a (Sex [*p* = 0.068], Diet [*p* = 0.0081], Interaction [*p* = 0.041]); Got1 (Sex [*p* = 0.0080], Diet [*p* = 0.67], Interaction [*p* = 0.92]); Mdh1 (Sex [*p* = 0.0029], Diet [*p* = 0.18], Interaction [*p* = 0.61]); Scl25a11 (Sex [*p* = 0.0031], Diet [*p* = 0.0054], Interaction [*p* = 0.026]). Sample sizes for each analysis provided in Supplemental Table S3

Sex differences similarly accounted for most changes in the abundance of cartilage metabolic proteins (Fig. 7A). Out of 101 targeted proteins, HFD or sex altered 41 proteins (*p* < 0.05), including 20 that only differed by sex. Many sex differences were characterized by greater protein abundance in female versus male cartilage, as seen in clusters 1 and 4 (Fig. 7A, Supplemental Table S5). We performed a KEGG enrichment pathway analysis based on the proteins in each cluster, and clusters 1 and 4 were enriched for proteins involved in glycolysis/gluconeogenesis and biosynthesis of amino acids (Fig. 7B). In support of this analysis, we noted that many glycolytic proteins were more abundant in female versus male mice (Fig. 7C). In addition, proteins involved in the malate-aspartate shuttle were also more abundant in female mice. In contrast, cluster 3 proteins were generally more abundant in male mice, especially under HFD conditions (Fig. 7A). These proteins were enriched for the peroxisome proliferator-activated receptors (PPAR) signaling pathway, and consistent with this analysis, HFD increased the abundance of Cd36, Fabp4, and Cpt1a in male but not female mice (Fig. 7B, C). Thus, these data suggest that cartilage metabolism is sexually dimorphic at baseline and in response to HFD.

### Sexually dimorphic chondrocyte metabolism

**Fig. 8:**
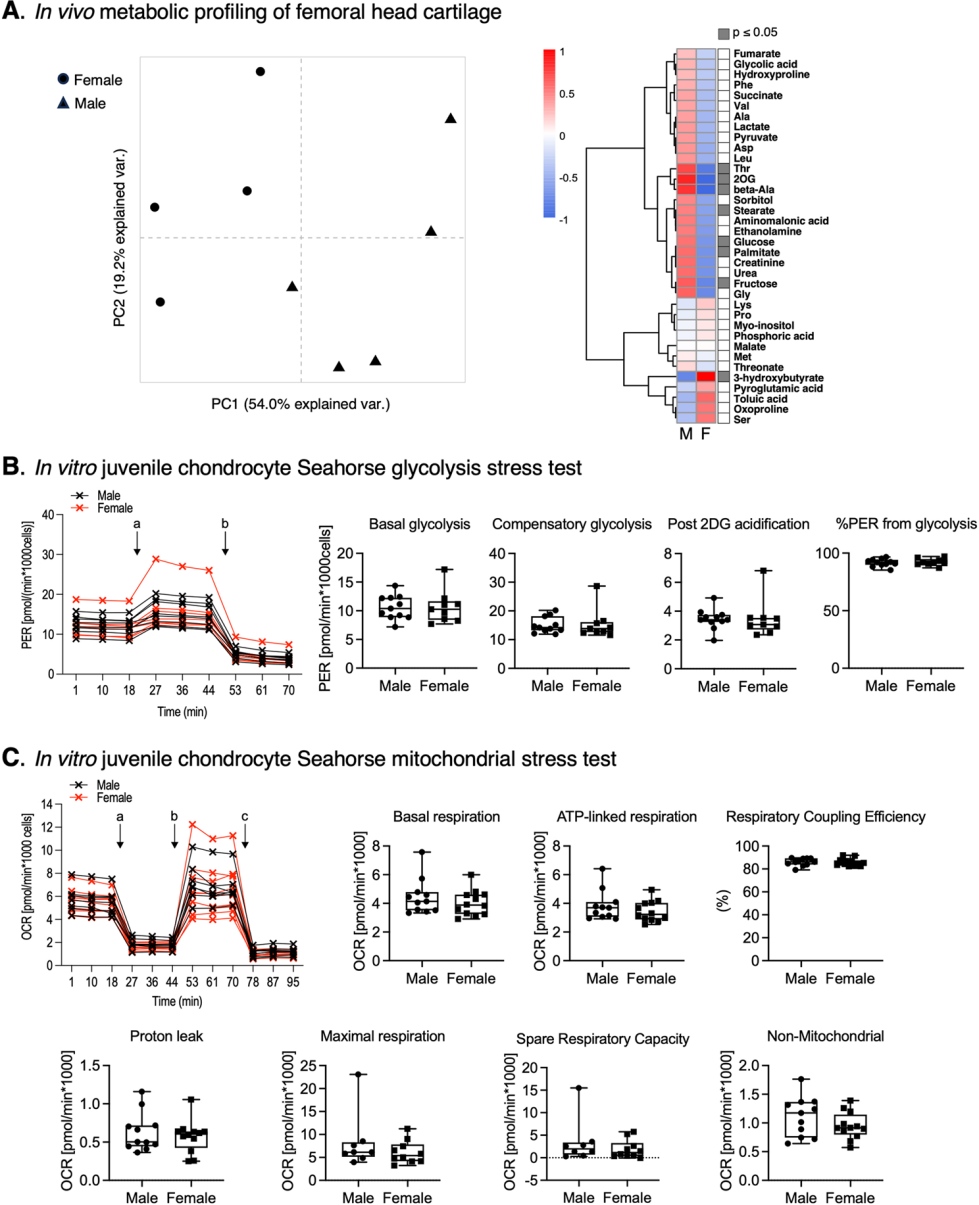
Evaluation of sex differences in chondrocyte metabolism from young, healthy animals prior to pubertal maturation. **A**In vivo metabolic profiling of femoral head cartilage from 5-week-old male and female mice by GC-MS [26]. Hip cartilage was used rather than knee cartilage because it enables rapid collection of sufficient cartilage tissue required for in vivo metabolic profiling. Left panel shows PCA results based on metabolite abundance values. Each point is an independent biological replicate consisting of cartilage pooled from 4 animals. Note the separation between female and male samples. Right panel shows the corresponding heatmap of metabolite abundance values following Z-transformation (mean = 0, stdev = 1) (M = male, F = female). Metabolite clustering based on Euclidean distance dissimilarity matrix. Adjacent column indicates significance based on Wilcoxon 2-sample test (*p* ≤ 0.05). Most differences were due to greater metabolite abundance in cartilage from male versus female mice. **B** Representative proton efflux rate (PER) data for Seahorse XF Glycolytic Rate Assay comparing passage one primary juvenile chondrocytes isolated from knee epiphyseal cartilage of 6 to 8-day old male (black) and female (red) mice tested on the same plate. Each “x” symbol is the average value of technical duplicates, and lines connect data from the same donor. “a” indicates addition of Rot/AA and “b” addition of 2-DG, as per manufacturer protocol. Boxplot panels show summary of Glycolytic Rate Assay results comparing male and female cells. *p* > 0.10 for all comparisons (Student’s t-test). **C** Representative oxygen consumption rate (OCR) data for Seahorse XF Cell Mito Stress Test Assay comparing primary juvenile chondrocytes isolated from male (back) and female (red) mice tested on the same plate. “a” indicates addition of oligomycin, “b” addition of FCCP, and “c” addition of Rot/AA, as per manufacturer protocol. Boxplot panels show summary of Mito Stress Test Assay results comparing male and female cells. *p* > 0.10 for all comparisons (Student’s t-test)

We next tested for intrinsic sex differences in chondrocyte metabolism from young, healthy animals prior to pubertal maturation using in vivo metabolic profiling of hip cartilage from 5-week-old male and female mice. Hip cartilage was used rather than knee cartilage because it enables sufficient tissue to be rapidly collected [26], which is critical for in vivo metabolic profiling. PCA and hierarchical cluster heat map analyses indicate sex differences in cartilage metabolite abundance (Fig. 8A). Most differentially abundant metabolites were greater in males, including sugars (glucose, fructose), amino acids (threonine, beta-alanine), and lipids (palmitate, stearate). In contrast, 3-hydroxybutyrate was more abundant in cartilage from female mice. To test for differences in chondrocyte metabolism independent of the in vivo environment, we isolated primary juvenile chondrocytes from 6-8-day-old mice and tested passage 1 cells using the Seahorse XF glycolytic rate and mitochondrial stress test assays. No differences were observed in the rate of basal glycolysis, compensatory glycolysis, or the percent of proton efflux rate attributed to glycolysis (Fig. 8B). Similarly, we also did not observe any sex differences in mitochondrial respiration parameters, including basal, ATP-linked, or maximal oxygen consumption rates (Fig. 8C). These data suggest that the in vivo environment is important for inducing sex differences in chondrocyte metabolism.

## Discussion

Obesity is a major risk factor for knee OA, with recent evidence indicating that the effect size is greater in women than men [5–7]. We explored potential sex differences in how obesity increases OA risk using a mouse model of HFD-induced obesity. We tested the hypothesis that 20 weeks of HFD would induce sexually dimorphic changes in both systemic and local risk factors of knee OA. Male and female mice developed mild cartilage pathology and synovial hyperplasia in response to HFD, although only male mice developed osteophytes. Systemically, HFD treatment induced a combination of concordant and discordant changes in metabolism and the gut microbiome between male and female mice. Locally, though, most HFD effects were discordant between sexes, suggesting that OA pathophysiology is distinct between sexes during the early phase of disease. It is important to note that HFD-induced OA pathology was less severe in female versus male mice, which is opposite of the heightened OA risk observed in women versus men with obesity. Thus, in the following sections we discuss the findings in relation to the human literature to help evaluate the potential translational significance of our study.

One of the most notable sex differences was the elevated expression of inflammatory genes in synovial-IFP tissue with HFD versus Chow in female mice compared to male mice. Genes involved in monocyte/macrophage proliferation, differentiation, and recruitment, such as *Csf1*, *Klf6*, and *Ccr2*, were upregulated with HFD in female but not male mice. Additional genes that were similarly upregulated with HFD in female mice included those associated with neutrophil trafficking (*Cxcl1*), mesenchymal and hematopoietic progenitor cells (*Ly6a*,* Cd34*), fibrosis (*Col3a1*,* Ecm1*,* Adam17*), and tissue growth (*Ccn2*,* Tgfb1*,* Ngf*). Although we cannot exclude the possibility that inflammation had already resolved in male mice, the broad sex-dependent effects suggest that sustained synovium-IFP remodeling and inflammation are key features of HFD-induced OA in female mice. It is interesting to note that clinical data also suggests that joint inflammation contributes to knee OA in a sex-dependent manner as Hoffa-synovitis, determined by magnetic resonance imaging, increased the odds of developing incident radiographic knee OA in overweight women but not men [27].

Conversely, and consistent with previous reports [9], we observed a greater number of systemic metabolic disorders with HFD treatment in male versus female mice. For example, male mice but not female mice developed glucose intolerance and elevated serum glycerol, amylase, and numerous amino acids with HFD versus Chow. Liver triglycerides and serum alanine aminotransferase (ALT) were also elevated to a greater extent with HFD in male mice compared to female mice, consistent with greater HFD induction of hepatic steatosis in male mice [28]. In addition, serum cholesterol was more highly elevated in male mice, whereas taurine, a semi-essential amino acid with beneficial metabolic actions [29], was greater overall in female mice. Considering the growing evidence for a “metabolic OA” phenotype involving obesity, dyslipidemia, and hyperglycemia [8, 30, 31], systemic metabolic disorders may be stronger contributing factors to HFD-induced knee OA in male versus female mice. OA pathology is substantially greater in male versus female STR/Ort mice, which also exhibit sexually dimorphic systemic and local features of metabolic OA [32]. It is worth noting, though, that causal links between specific serum metabolic biomarkers and OA risk have yet to be clearly established [33]. Furthermore, as recent preclinical studies targeting cholesterol [34] and glucose [35] regulation have illustrated, metabolic interventions that modify one aspect of OA (e.g., cartilage pathology) do not necessary modify other aspects to an equal degree (e.g., synovitis or osteophytes) [36]. Thus, while there is considerable evidence that joint tissues function as an integrated organ unit, each joint tissue may be distinctly sensitive to different metabolic stressors.

We evaluated the tissue-specific effect of HFD on sex differences in OA risk by quantifying differences in gene expression and protein abundance in cartilage. KEGG enrichment pathway analysis of metabolic proteins revealed distinct sex differences and sexually dimorphic effects of HFD. Notably, proteins that clustered based on upregulation by HFD in female mice and downregulation in male mice were enriched for glycolysis, amino acid biosynthesis, and fructose/mannose metabolic pathways (Cluster 1, Fig. 7B). In contrast, proteins that clustered based on upregulation with HFD in male mice were enriched for the PPAR signaling pathway (Cluster 3, Fig. 7B), suggesting a sexually dimorphic upregulation of cartilage lipid metabolism in male mice. Together, these findings indicate key sex differences in cartilage metabolism under HFD conditions that cause early-stage OA. OA cartilage has been characterized by a multitude of metabolic changes [8, 37], including impaired glycolysis and glucose uptake [38], upregulated glycolysis [39, 40], impaired oxidative phosphorylation [41–43], and altered lipid metabolism [24, 44–47]. Our findings suggest that sex differences may contribute to the different metabolic phenotypes observed in OA cartilage.

Recent analyses of post-injury synovial fluid metabolites [48] and chondrocytes isolated from end-stage OA knees undergoing arthroplasty [49] indicate that sex differences in cartilage metabolism are also present in humans. However, unlike the findings from mice, OA chondrocytes isolated from male patients were characterized by greater glucose uptake and reliance on glycolysis for cellular energy production compared to cells from female patients; whereas female OA chondrocytes were characterized by greater lipid uptake and oxidative phosphorylation compared to male patients’ cells [49]. The basis for the observed sex differences in metabolic phenotypes between mice and humans remains to be determined. While most human OA cells were obtained from overweight and obese patients (median body mass index of ~ 28 kg/m^2^) [49], the study did not compare cells obtained from patients with normal versus obese body mass indexes to evaluate differences due to obesity. The different stages of disease as well as the drivers of OA may further confound the comparison between human data and the current mouse study, as human chondrocytes were obtained from older adults with end-stage OA and mouse cartilage was obtained from adult animals with obesity-induced early-stage OA. Chondrocyte metabolism varies by cartilage zone, and therefore, tissue loss due to advanced OA progression or species-related differences in zone phenotypes may further confound human and mouse comparisons. There are additional challenges for comparing chondrocyte metabolism and sexually dimorphic OA risk, such as joint specificity (e.g., knee versus hip), potential epigenetic contributions (e.g., due to age, hormone exposure, or mechanical loading), and differences in metabolites measured under fresh flash-frozen versus in vitro culture conditions [26]. Taken together, these observations indicate the need for further study to understand how sex differences in chondrocyte metabolism may contribute to sex-dependent OA pathophysiology. Given that our in vitro analysis of cellular metabolism in primary juvenile chondrocytes did not indicate intrinsic sex differences (Fig. 8), it appears that chondrocyte-extrinsic factors likely contribute to the observed sex differences in chondrocyte metabolism.

While recent reviews of clinical [4, 5] and preclinical [50] research have highlighted critical knowledge gaps about sex-specific OA risk factors, there has been progress. In preclinical studies of post-traumatic knee OA, female mice develop less severe structural pathology than male mice [51–54], thereby providing an opportunity to study the basis for this sex difference. A potential reason for the reduced pathology in female mice is that inflammation is more attenuated [54], which is mediated in part through sex differences in the gut microbiome [55]. An additional factor may be heightened pain sensitivity in female mice that protects against excessive joint loading [52]. Sex differences are also present in age-associated knee OA, with older male and female mice developing worse mechanical allodynia and knee hyperalgesia compared to younger mice despite less severe OA pathology in older female versus male mice [56]. Intriguingly, aging amplified sex differences in inflammatory markers in the dorsal root ganglion [56].

In the context of obesity, changes in gut microbiota composition, serum lipopolysaccharide (LPS), and altered microbial metabolism may be pivotal mediators of the observed sex differences in OA. Gut microbiota composition and gut permeability to inflammatory mediators, such as LPS, are increasingly recognized as OA risk factors [20, 57–61]. We recently reported a comparison of male and female C57BL/6J gut microbiota composition and demonstrated that cross-sex gut microbiome transplantation can significantly alter OA outcomes after meniscal injury [20]. Despite using a different taxonomy reference set, several sex-specific clades overlap with the present study; specifically, members of genus *Aldercreutzia* within class *Coriobacteriia* and genus *Lactobacillus* are characteristic of the female microbiome, whereas class *Clostridia* is enriched in male mice. We also recently completed an analysis of both cecal and cartilage microbial patterns in young, aged, and HFD-treated mice [62]. The cecal data from the current study showed similar microbiome shifts with HFD, including decreases in phylum *Actinobacteria* and family *Burkholderiales* and increases in family *Staphylococcaceae*, among others.

Our data also demonstrate several overlaps with a previous study identifying changes in the gut microbiome and increased OA risk with HFD in male mice, which was ‘rescued’ via dietary supplementation with oligofructose fiber [60]. The key microbial species that was reduced in HFD and increased with oligofructose was *Bifidobacterium pseudolongum*. In the present study, members of *Bifidobacterium* were less abundant with HFD versus Chow, although this diet effect was primarily observed in male mice (e.g., *B*. *pseudolongum* sub-species *globosum*). Moreover, *Bifidobacterium* was more abundant in female versus male mice both on Chow and HFD. Conversely, phylum *Firmicutes A* members were more abundant in male versus female mice under Chow diet but were substantially greater in female mice under HFD conditions. While some family members, such as *Peptostreptococcaceae*, were more abundant with HFD in both sexes, *Lachnospiraceae* family members were only more abundant with HFD in female mice. The *Lachnospiraceae* clade has been previously associated with inflammatory autoimmune diseases, including type 1 diabetes mellitus [63] and preclinical rheumatoid arthritis [64]. Furthermore, three clades that were significantly altered by HFD also demonstrated sex-specific differences, including enrichment in females in genus *Alistipes* and enrichment in males in genera *Lactococcus* and *Streptococcus. Alistipes* was positively associated with OA in a recent analysis of microbiome data from the UK Twins study [65], and *Streptococcus* was associated with pain in human knee OA patients [66].

Despite these sex differences in gut microbiota composition, a recent case-control clinical analysis of altered gut microbiota and obesity-associated OA supports a link to increased intestinal permeability and elevated serum LPS rather than gut dysbiosis per se [61]. However, the OA patient cohort included a significantly higher percentage of women than the control cohort [61]. Thus, it remains to be determined if sexually dimorphic changes in gut microbiota or intestinal permeability contribute to a greater effect of obesity on OA risk in women versus men.

### Perspectives and significance

Our findings, together with the studies previously mentioned, further support a role for sex-specific biological processes mediating the effect of obesity on the risk of developing knee OA. Much work is needed, though, to elucidate the sex-specific pathways that modify the onset and progression of OA and to understand the translational significance of findings in mice to humans. For example, recent studies have highlighted a critical role for sex hormones in mediating HFD-induced metabolic disorders via changes in the gut microbiome and gut permeability [67, 68]. Similar approaches could be useful for identifying sex-dependent mechanisms linking obesity and OA, including gonadectomy, hormone replacement, and estrogen/androgen receptor genetic mutation and/or agonist treatment [9]. Another consideration is the potential contribution of the estrous cycle to outcomes within female mice. We did not monitor or control for the estrous cycle of the female mice tested in the current study, which could contribute to more variable outcomes as the estrous cycle influences the regulation of inflammatory gene transcription [69]. In addition, although we did not observe sex differences in the metabolism of primary immature chondrocytes isolated from knee epiphyseal cartilage of 6 to 8-day old mice, cell-intrinsic sex differences could contribute to OA risk through other mechanisms, including bone remodeling [70], macrophage activation [71], and intergenerational epigenetic programming [72]. An additional limitation of the current study is that sex differences in these or other potential mediators of OA were not evaluated prior to HFD treatment for a more rigorous evaluation of sex and diet differences. Furthermore, while we have previously reported on HFD-induced pain behavior and functional impairment in male and female mice [15, 16], those studies were performed separately using different diets and experimental durations. Given the reported sex differences in pain and disease severity in rodent models of post-traumatic OA [73], a similar comparison should be conducted for obesity-associated OA.

By focusing on sex-differences in early-stage OA using a HFD-induced model of obesity, this research is significant based on the current perspective that treating OA early offers a greater chance of success in preventing disease progression than waiting until a more advanced stage of disease [74, 75]. We found that most effects of HFD on joint tissues were discordant between sexes, suggesting that OA pathophysiology is distinct between sexes during the early phase of disease. Understanding the molecular pathways that are activated during early disease is critical for developing effective OA therapies, especially if different strategies are required for men and women. Moreover, these findings may help inform how sex differences contribute to other rheumatic diseases that are associated with metabolic disorders.

## Electronic supplementary material

Below is the link to the electronic supplementary material.

Supplementary Material 1

Supplementary Material 2

## Data Availability

Data that support the findings of this study are available from the corresponding author upon reasonable request.

## Abbreviations

GC-MS: Gas-chromatography mass-spectrometry
HFD: High fat diet
IACUC: Institutional Animal Care and Use Committee
IFP: Infrapatellar fat pad
LDA: Linear discriminate analysis
OA: Osteoarthritis
OARSI: Osteoarthritis Research Society International
QMR: Quantitative magnetic resonance
RER: Respiratory Exchange Ratio
SRM: Selective reaction monitoring
