# Supplementary material for: Sexually dimorphic metabolic effects of a high fat diet on knee osteoarthritis in mice
Source: Biol Sex Differ. 2024 Dec 5;15:103. doi: 10.1186/s13293-024-00680-6 (PMC11619521; doi:10.1186/s13293-024-00680-6)
Supplement: Supplementary file 2 — Supplementary Material 2 [file 13293_2024_680_MOESM2_ESM.docx]

**Supplemental Materials**

**Sexually dimorphic metabolic effects of a high fat diet on knee osteoarthritis in mice**

Contents:

Supplemental Results

- p2: Table S1. 96.96 IFC Array Custom DELTAgene Target List for IFP-Synovium Sample Analyses
- p3: Table S2. 96.96 IFC Array Custom DELTAgene Target List for Cartilage Sample Analyses
- p4: Table S3. Summary of Sample Sizes for Analyses
- p5: Figure S1. MetaboAnalyst KEGG Pathway Analysis of Sex-Specific Effects of HFD on Serum Metabolites
- p6: Table S5. Cartilage Protein Abundance Data

Note: Gut microbiome Supplemental Table S4a-x provided as separate excel file attachment

**Table S1.** 96.96 IFC Array Custom DELTAgene Target List for IFP-Synovium Sample Analyses

Genes evaluated by quantitative RT-PCR as shown in Figure 4. Primers were obtained using the Delta Gene qPCR Gene Expression D3 Assay Design Program (Fluidigm), which is MIQE compliant and optimized for the Biomark HD system.

**Table S2.** 96.96 IFC Array Custom DELTAgene Target List for Cartilage Sample Analyses

Genes evaluated by quantitative RT-PCR as shown in Figure 6. Primers were obtained using the Delta Gene qPCR Gene Expression D3 Assay Design Program (Fluidigm), which is MIQE compliant and optimized for the Biomark HD system.

**Table S3. Summary of Sample Sizes for Analyses**

Sample sizes were based on power analyses for OA histopathology, our primary outcome. Using data from prior HFD studies in our lab, n=9 animals per group was estimated to provide 80% power to detect a 30% difference in mean OARSI scores with a significance level of p=0.05 (mean OARSI score of 1.0 and standard deviation of 0.2). We tested n=12 per group in case of unexpected animal health or tissue processing issues. No animals died prior to study completion, although some outcomes included n<12 per group due to quality control (QC) related technical issues with sample processing, insufficient sample availability or detection, or assay sample capacity limitations, as summarized below.

**Figure S1. Sex-Specific MetaboAnalyst KEGG Pathway Analysis of HFD Effect on Serum Metabolites.** HFD altered the relative abundance of 25/49 serum metabolites measured by GC-MS, with most differences observed in males (22/49) and half as many occurring in females (11/49). We used the MetaboAnalysts online Pathway Analysis tool to perform a Fisher's exact test by KEGG *Mus musculus* database for altered metabolites. Significantly affected pathways were chosen based on FDR-corrected q-values < 0.10. Pathway scatter plots show pathway impact scores (x-axis) and −log10(P) values of enrichment analysis results (y-axis). Impact scores represent normalized topology measures of perturbed metabolites in each pathway. Data point sizes correspond to pathway impact values, and the color gradient corresponds to the enrichment p-value.

**Table S5.** Cartilage Protein Abundance Data

Target proteins organized corresponding to heatmap shown in Figure 7A. Values represent protein abundance in cartilage based on the geomean of peptide areas, normalized to a stable reference protein, Hspd1.

**Table S5. (cont.)**
